# Relaxation–Diffusion T2–ADC Correlations in Breast Cancer Patients: A Spatiotemporally Encoded 3T MRI Assessment

**DOI:** 10.3390/diagnostics13233516

**Published:** 2023-11-23

**Authors:** Martins Otikovs, Noam Nissan, Edna Furman-Haran, Debbie Anaby, Ravit Agassi, Miri Sklair-Levy, Lucio Frydman

**Affiliations:** 1 Department of Chemical and Biological Physics, Weizmann Institute of Science, Rehovot 7610001, Israel; 2 Department of Radiology, Sheba Medical Center, Ramat Gan 5262000, Israel; noamniss@gmail.com (N.N.); debbie.anaby@sheba.health.gov.il (D.A.); miri.sklairlevy@sheba.health.gov.il (M.S.-L.); 3 Sackler School of Medicine, Tel-Aviv University, Tel Aviv 6997801, Israel; 4 Life Sciences Core Facilities, Weizmann Institute of Science, Rehovot 7610001, Israel; edna.haran@weizmann.ac.il; 5 Azrieli National Center for Brain Imaging, Weizmann Institute of Science, Rehovot 7610001, Israel; 6 Surgery Department, Soroka Hospital, Beer Sheva 8410101, Israel

**Keywords:** breast cancer, diffusion-weighted MRI, T2 measurements, cancer diagnosis

## Abstract

Quantitative correlations between T2 and ADC values were explored on cancerous breast lesions using spatiotemporally encoded (SPEN) MRI. To this end, T2 maps of patients were measured at more than one b-value, and ADC maps at several echo time values were recorded. SPEN delivered quality, artifact-free, TE-weighted DW images, from which T2-ADC correlations could be obtained despite the signal losses brought about by diffusion and relaxation. Data confirmed known aspects of breast cancer lesions, including their reduced ADC values vs. healthy tissue. Data also revealed an anticorrelation between the T2 and ADC values, when comparing regions with healthy and diseased tissues. This is contrary to expectations based on simple water restriction considerations. It is also contrary to what has been observed in a majority of porous materials and tissues. Differences between the healthy tissue of the lesion-affected breast and healthy tissue in the contralateral breast were also noticed. The potential significance of these trends is discussed, as is the potential of combining T2- and ADC-weightings to achieve an enhanced endogenous MRI contrast about the location of breast cancer lesions.

## 1. Introduction

Diffusion-weighted imaging (DWI) can increase the diagnostic value provided by dynamic contrast-enhanced (DCE) MRI for breast cancer characterizations [1] and, as such, it is rapidly becoming an integral part of the multiparametric breast cancer MRI protocol [2]. DWI relies on the intrinsic contrast provided by breast tumor masses on the apparent diffusion coefficient (ADC [3,4]) of water molecules in breast cancer, which experiences a significant reduction vs. its value in healthy fibroglandular tissue. This is assumed to reflect an increased local cellularity of the tumoral masses [5,6], which, by either increasing the macromolecular protein/membranous content and/or by reducing the available extracellular space, reflects a restricted water diffusivity. An increase in macromolecular density should also affect the transverse relaxation time, T2, of the water [7]. T2-weighted images do indeed play a role in the breast cancer MRI analysis, by providing an anatomical reference [8], improving the delineation of non-mass ductal carcinoma in situ (DCIS) lesions [9], and omitting unnecessary biopsies when identifying benign, bright lesions in T2-weighted scans [10,11,12]. However, beyond serving as an adjunct tool to better characterize the breast anatomy, the use of quantitative T2 values is uncommon in the clinical settings—even if its potential benefits for breast imaging have often been discussed [13,14,15]. This stems from various reasons, including long scan times associated with the acquisition of quantitative T2 information, as well as relatively low levels of separation between benign and malignant tissues when using solely T2 [16].

Given the fact that cell density and macromolecular content could influence both water’s transverse relaxation and its spatial diffusivity, the question arises whether insight about the physical influence of the former factors could be gained by correlating T2 and ADC values exhibited throughout healthy and tumoral regions—not as averages within cohorts, but on a one-on-one basis. Indeed, this type of 2D-like correlation has been shown useful for untangling common factors relating these properties in soft, porous, and living matter [17,18,19]; it might thus also serve for clarifying matters in complex tissues such as breast and conceivably help improve the contrast provided by either quantitative T2 or ADC maps, separately. This approach was in fact explored in prostate MRI studies, which concluded that associating ADC and T2 values could have potential for improving the diagnostic accuracy versus cases when ADC or T2 maps alone were used [20,21,22]. While the combined use of T2- and diffusion-weighted images has been reported to improve breast cancer diagnosis [23,24], to the best of our knowledge, the potential of quantitatively combining the values associated with ADC and T2 maps has not yet been assessed in breast imaging. Analyzing such T2–ADC correlations demands acquiring T2 maps at more than one diffusion-encoded b-value, and ADC maps at more than one T2-encoding echo time (TE) value. Performing such nested T2–ADC mappings results in an increased data dimensionality and extended scan times. This, in turn, brings additional challenges in terms of image inconsistencies in phase and amplitude, both as a consequence of patient motion during the longer scan times, and as a result of the weighting imposed by the diffusion-encoding gradients. To address these challenges, T2 mapping in the presence of diffusion-encoding gradients is usually executed using echo-planar imaging (EPI) readouts [20]; studies have validated the consistency of the T2 parameters thus derived with those arising from multi-spin-echo sequences, even when diffusion-sensitizing gradients are present [20,25].

EPI-based methods still suffer from limitations, including distortions along the phase-encoding (PE) direction as a consequence of B_0_ field inhomogeneities [26], as well as complications when having to deal with coexisting fat and water signals in the breast [27]. These challenges, as well as added hurdles arising from breathing and cardiac motions, were present in the cases on which this study focused. To bypass these limitations, we explored here the application of spatiotemporally encoded (SPEN) MRI [28,29,30]. SPEN has a demonstrated ability to deliver quality single-shot and segmented human breast DWI maps, by relying on superior T2* refocusing properties [31,32], and on the possibility of performing a self-referenced motion correction along the PE direction [33,34]. This has helped to collect high-resolution, strongly b-weighted ADC maps in a number of breast-oriented clinical studies [35,36,37,38]. The present work exploits this potential by suitably modifying SPEN-based sequences, so as to deliver T2–ADC correlated maps. Data obtained in a 12-patient breast cancer cohort confirmed a statistically significant ability of ADC to distinguish malignant from normal tissue; although this discrimination was absent along the “T2 axis”, a weak but consistent anticorrelation emerged upon comparing the ADC and T2 values. Indeed, tumorous regions whose waters had consistently lower diffusivities also exhibited consistently longer relaxation times than either surrounding healthy and/or contralateral breast tissue. This was somewhat unexpected, as constrained water diffusivity would normally be associated with higher cellular or macromolecular densities of the kind one would also expect to shorten T2 times. Potential reasons behind this seeming contradiction are put forward, and conclusions emerging from these measurements about optimized DWI measuring parameters for an improved tumor contrast are discussed.

## 2. Methods

**Patients.** The study was approved by the Internal Review Boards (IRB) of the Sheba Medical Center (Ramat-Gan, Israel), and a signed informed consent was obtained from all subjects. Twelve breast cancer patients (mean age 44, range 27 to 66 years old) and one healthy volunteer (33 years old) were prospectively enrolled in this study. Clinical characteristics of the breast cancer cohort are summarized in Table 1.

**Figure 1:**
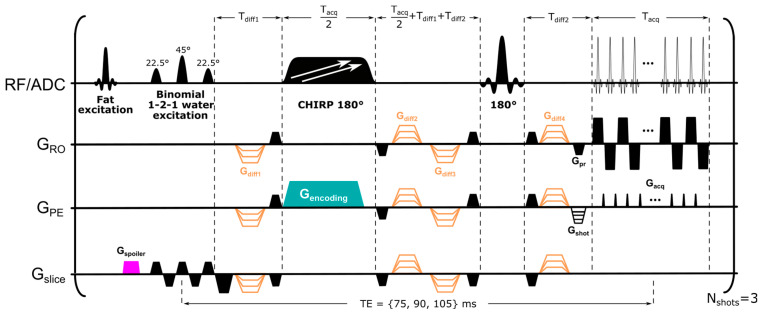
SPEN sequence used in this study, employing dual frequency swept chirp pulses for simultaneous spatial encoding of two frequency ranges along the PE direction. The sequence incorporates diffusion-sensitizing gradients (in orange). To minimize intensity of residual fat signal, a fat-excitation/spoiler unit, followed by a slice-selective binomial 1-2-1 water excitation pulse, was used. Diffusion gradient timings were calculated as described by Reese et al. [42]. Abbreviations: G_spoiler_, spoiler gradient; G_encoding_, encoding gradient; G_slice_, slice-selective direction; G_PE_, phase-encoding direction, corresponding to direction of SPEN encoding; G_RO_, readout direction; G_pr_, readout prephase gradient; G_shot_, phase-encoding prephase gradient; G_acq_, blipped acquisition gradients; T_diff_1_ and T_diff_2_, duration of first and last diffusion-encoding gradients; T_acq_, acquisition time; N_shots_, number of interleaved shots (segments). For acquiring the coil sensitivity maps, the dual frequency swept chirps were replaced with a single chirp sweeping a single-breast frequency range, while the rest of the parameters remained unchanged.

**Pulse sequence: General considerations.** SPEN is an ultrafast imaging alternative, offering better distortion reduction than EPI when facing inhomogeneous magnetic fields. SPEN achieves this by controlling the effective PE bandwidth over the course of the excitation, via the action of a frequency swept (chirped) pulse encoding acting while in the presence of a gradient [28,32]. This leads to a quadratic phase profile possessing a sensitive focal point at its vertex, that can then be moved along the targeted PE field-of-view (FOV) by the action of an acquisition gradient. This operation “rasterizes” the image, allowing one to collect the imaging data while refocusing the T2* effects for every position along the PE acquisition axis; notice that this is unlike a normal spin echo behavior, where T2* refocusing occurs only at the center of k-space. Such “fully refocused” SPEN MRI version was implemented here as introduced in ref. [32], with a sequence modified to incorporate multiband excitation. This involved applying two linear, chirped encoding pulses acting simultaneously over two frequency ranges [39], with each of these covering (encoding) one breast. This information was subsequently unraveled by multiple receiving [36]. Such dual sweeping proved advantageous in terms of minimizing the TE compared to alternative single-sweep options that skip k-space lines and rely on parallel receivers to deliver full resolution images [40]; this is because, in the latter case, targeting an FOV that would include both breasts would also require scanning the void, information-less region in-between them. The resulting pulse sequence—which, in addition to full refocusing and dual-band excitation over the two breasts, also incorporated interleaved multi-segment acquisitions along the PE direction—is presented in Figure 1. To ensure more robust fat suppression, this sequence also used a frequency-selective fat excitation/spoiling gradient unit, followed by a water- and slice-selective excitation pulse, composed of a binomial 1-2-1 pattern, to minimally perturb any unsuppressed fat signal [41]. The use of a water-selective excitation also allowed us to suppress the resonances arising from silicone implants, which was relevant for one of the patients enrolled in the study. All these provisions enabled us to cover both breasts with a signal-to-noise ratio (SNR) that could sufficiently perform correlated ADC and T2 mappings, and thus explore the cellular environment based on complementary diffusivity/transverse relaxation properties.

**SPEN image reconstruction.** Image reconstructions relied on sensitivity maps accounting for the different signals received by each coil, which were collected in two separate scans using the SPEN sequence described above, with each scan targeting one of the breasts. This was carried out by applying just one of the sweeps used in the dual-band encoding in Figure 1, while setting the diffusion-encoding gradients to zero. Assuming that the chirped pulse addressed two bands, *b*_1_ and *b*_2_, whose images are being sought, and denoting the signal acquired in each of interleaved shot, *m* as *y*(*m*), we can then write the full image reconstruction as

(1) $$y\left(m\right)=\left[\begin{array}{cc} A_{m,1} & A_{m,2} \end{array}\right]\left[\begin{array}{cc} S_{1} & S_{1}\\ S_{2} & S_{2} \end{array}\right]\left[\begin{array}{c} b_{1}\\ b_{2} \end{array}\right]$$

where *S*_1_ and *S*_2_ are the coil sensitivity maps corresponding to the respective bands, and {*A_m,_*_1_, *A_m,_*_2_} are the matrices to be used in the super-resolution (SR) processing of the data [43] for shot *m* and band *i* = 1, 2. After collecting the *N_shot_* signals ${\left\{y(m)\right\}}_{m=1,N_{shot}}$, where *N_shot_* is the number of acquired interleaves along the PE direction, the image reconstruction problem was recast as

(2) $$arg\;\underset{x}{\min}\frac{1}{2}{\left\|y-A\cdot S\cdot x\right\|}_{2}^{2}+\lambda {\left\|\Psi x\right\|}_{2}$$

where *y* corresponds to the combination of all acquired signals y(m), $x={\left[\begin{array}{cc} b_{1} & b_{2} \end{array}\right]}^{T}$ are the images being sought after combination of the simultaneously excited bands, ***S*** corresponds to the coil sensitivity maps, ***A*** is the SR operator including the effects of SPEN’s quadratic phase encoding, *λ* is a regularization parameter, and **Ψ** denotes a finite difference operator. This reconstruction still requires accounting for motional and even–odd echo effects—both relevant in breast diffusivity studies. To derive the even–odd phase corrections, interleaved shots were first combined together for data sets acquired without diffusion weighting, and two systems of equations were solved after recasting the problem in the form of Equation (2) while selecting for each system of equations either the “even” or the “odd” lines of k-space [34]. Following these two reconstructions, a “reference-less” even–odd phase correction of all data sets was obtained, based on the phase differences among these two images. Finally, the motion between shots that may corrupt the diffusion-weighted data was accounted for by including the possibility of phase changes Φ*_m,i_*. caused by patient movement and affecting the images of shot *m* and band *i*. This was carried out by modifying Equation (1) into

(3) $$y\left(m\right)=\left[\begin{array}{cc} A_{m,1} & A_{m,2} \end{array}\right]\left[\begin{array}{cc} S_{1} & S_{1}\\ S_{2} & S_{2} \end{array}\right]\left[\begin{array}{cc} \Phi_{m,1} & 0\\ 0 & \Phi_{m,2} \end{array}\right]\left[\begin{array}{c} b_{1}\\ b_{2} \end{array}\right]$$

This equation was solved after recasting it once again as a minimization problem, using the aforementioned coil sensitivity maps and even–odd phase correction information, and then solving each complex per-shot image corresponding to bands 1 and 2. This delivered motion-compensated phase maps Φ*_m,i_*, which were incorporated into the sensitivity maps as *Scor_m,i_ =* Φ_m,*i*_*S_i_* for every shot and band; the full reconstruction problem was then solved once again following the formulation of Equation (2).

**MRI protocols.** Images were acquired at 3 T on a Siemens Prisma scanner using a 16-channel bilateral receiving breast coil. The clinical protocol consisted of anatomical reference images acquired using a T2-weighted two-dimensional turbo-spin-echo sequence covering the whole chest collected with a 1.1 × 0.9 × 2.0 mm^3^ image resolution, followed by DCE measurements carried out using a T1-weighted three-dimensional gradient echo (GRE) sequence that used Dixon fat suppression and covered both breasts at a 1.1 × 0.9 × 1.5 mm^3^ resolution. Gadoterate meglumine (Dotarem 0.5 M, Guerbet, France) was injected for these DCE measurements at 0.1 mmol/kg body weight, followed by 20 mL of saline flush (both at a rate of 2 mL/s). One pre-injection followed by seven post-injection image series were acquired, with 57 s required to record each of the series. The DCE images presented in this article were obtained by subtracting the pre-injection image from that arising in the second time point after administration of the contrast agent. The research portion of this study was acquired after the T2w acquisitions but before the DCE ones, to avoid potential influences of the contrast agent on the quantitative parameter mapping.

To further explore SPEN’s capability to overcome susceptibility-derived image distortions, a DWI series with comparable parameters to those in the SPEN acquisitions was also acquired for TE = 67 ms, using readout-segmented EPI (RESOLVE)—one of the most widely used approaches for performing DWI in regions suffering from B_0_ inhomogeneity [44]. SPEN and RESOLVE images were acquired in axial orientations at a 1.2 × 1.2 × 3.0 mm^3^ resolution using diffusion weightings (repetitions) of 0 (1) and 800 (3) s/mm^2^, and collected for three orthogonal diffusion directions using bipolar diffusion-encoding schemes. A Siemens-provided sequence was used to acquire these RESOLVE images, with an FOV of 300 × 180 (RO × PE) mm^2^, 3 readout (RO) segments, a GRAPPA acceleration factor of 2 along the PE dimension, an echo spacing of 0.5 ms, and an effective PE bandwidth (BW) of 4 kHz. In order to reduce the number of acquired PE lines and thus TE, RESOLVE’s phase-encoding direction was set as anterior-to-posterior; this provided the shortest FOV dimension capable of covering both breasts and a small portion of the chest, while folding was avoided by applying outer volume saturation. A total of 30–40 slices with a 50% gap between slices and a TR of 6.3 s were typically acquired, resulting in an acquisition time of 3 min 30 s.

After identifying the lesion’s location based on DW images and ADC maps, SPEN acquisitions were performed for 10 slices, with a 50% gap between the slices, using the sequence in Figure 1 and a TR of 12 s. SPEN images were collected for b = 0 and 800 s/mm^2^ nominal diffusion weightings at three different TEs (75, 90, and 105 ms), in order to derive T2–ADC correlations on a per-voxel basis. Each breast was covered with an FOV spanning 140 × 94 (RO × PE) mm^2^, simultaneously excited using a dual frequency swept chirp pulse. SPEN’s PE direction was set right-to-left, and three interleaved segments along this dimension were acquired. The time–BW product of the chirp pulses used was 40 and the echo spacing was 0.93 ms, resulting in an effective PE BW of 3.3 kHz. Each of the DW images in the series took 6 min, resulting in a total acquisition time for the combined T2–ADC mapping of ca. 19 min. While this could have been accelerated using multi-echo sequences [45,46], it would have resulted in a minimum TE step of 30 ms for the consecutive echoes, as well as in an SNR that was too low to obtain faithful T2–ADC maps. Sensitivity maps for each breast were acquired, as mentioned, using two separate, single swept chirped SPEN pulses, b = 0 s/mm^2^ and TE = 75 ms, but otherwise identical parameters to those used for the acquisition of the remaining experiments; these mappings lasted 36 s.

**Data analysis.** Before averaging over repetitions and diffusion directions, the SPEN images were denoised as described by Veraart et al. [47]. Arithmetic and geometric averages were subsequently calculated over repetitions and diffusion directions, respectively, for each TE and b-value. This was followed by a least-square fitting of the data,

(4a) $$S_{TE}\left(b\right)=S_{TE}\left(0\right)\cdot exp\left(-b\times {ADC}_{TE}\right)$$

to derive ADC values per each recorded TE, where $S_{TE}\left(b\right)$ and $S_{TE}(0)$ denote per-voxel signal intensities with and without diffusion encoding at a given TE, and ${ADC}_{TE}$ is the isotropic apparent diffusion coefficient at the respective TE. In addition, b-dependent T2 values were derived from

(4b) $$S_{b}\left(TE\right)=S_{b}\left(0\right)\cdot exp\left(-\frac{TE}{T_{2,b}}\right)$$

where $S_{b}\left(TE\right)$ corresponds to the image intensity in each voxel acquired with a given TE and b-weighting, $S_{b}\left(0\right)$ is a constant corresponding to the proton density weighted by T1, by diffusion and by the sequence parameters, and $T_{2,b}$ is the T2 value at the respective b-weighting.

The lesion and fibroglandular tissue borders were delineated by M.O. (6-year experience in breast imaging) based on DCE and DWI images, taking as an anatomical reference the T2-weighted data to exclude regions containing mainly signals from fatty or necrotic tissues. After performing the segmentation, average values over the corresponding regions were extracted using a Matlab script (The Mathworks, Inc., Natick, MA, USA). Box plots were generated by averaging overall values arising from, respectively, cancerous and healthy tissue, while also distinguishing between healthy tissue in the ipsilateral and contralateral breast. For healthy tissue, values were calculated only from those regions in ipsilateral and contralateral slices that had a cancerous counterpart, excluding from analysis those slices in which lesions were not identified. For performing the correlation plots, the average value for each tissue type was calculated per each slice, hence the number of points corresponds to the number of total analyzed slices per tissue type.

## 3. Results

**Figure 2:**
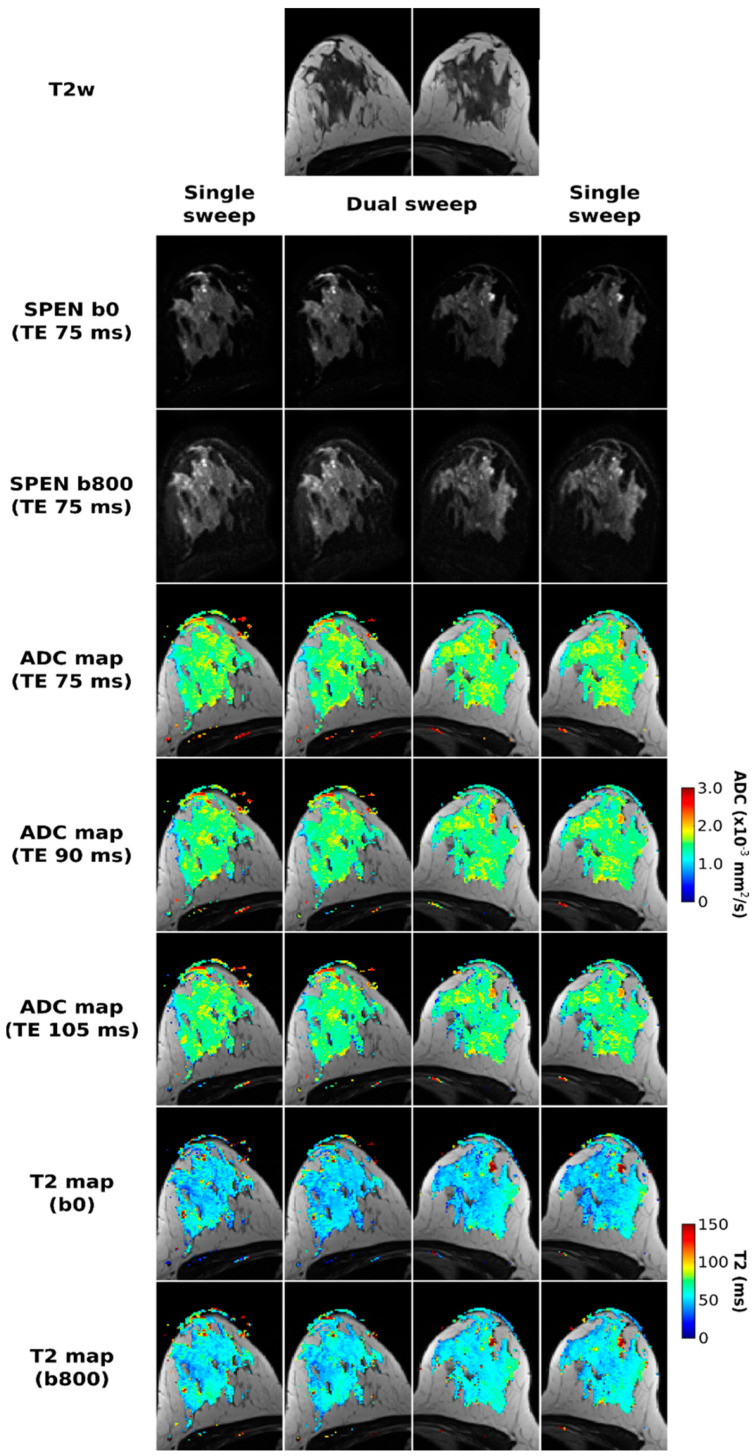
Comparisons between SPEN acquisitions performed using a single (right and left columns) and dual (two central columns) frequency swept chirp pulses. All experiments employed three interleaved shots along the PE dimension. SPEN b0 and b800 images acquired with TE = 75 ms are presented in the second and third row, respectively. In the following rows, ADC maps for each of the TEs used (75, 90, and 105 ms), as well T2 maps calculated for the two b-values used, are presented overlayed on the T2w images displayed in the first row.

Figure 2 presents a set of healthy volunteer results, collected to assess the overall methodology. The SPEN images in the two central columns correspond to acquisitions arising from various TE/b-value combinations with *N_shot_* = 3 interleaves and a dual-band chirp excitation simultaneously sweeping over the two breasts, while the left- and right-most columns correspond to separate, single-breast interleaved SPEN acquisitions. The images acquired using single- and dual-band excitations, as well their respective quantitative maps, are virtually identical; this highlights the robustness of the proposed strategy to reconstruct multiband data. ADC maps are derived from either of the acquisitions for all TE values; similarly, T2 maps derived from b = 0 and 800 s/mm^2^ images are of satisfactory quality, when compared against the anatomical images, and yield a range of T2 values expected for a healthy volunteer at 3T. For instance, Glover et al. reported T2s in the 54.4 ± 9.4 ms range for healthy fibroglandular tissue at 3T [48], while we observe 51.7 ± 11.9 ms for b = 0 and 56.2 ± 10.6 ms for b = 800 s/mm^2^ images. The healthy volunteer data also yields the same T2 parameters using single or dual sweeps; the slight difference between the T2 maps derived from the b = 0 and 800 s/mm^2^ acquisitions, could arise from a diffusion filtering that attenuates the contribution of rapidly diffusing signal components when b = 800 s/mm^2^.

**Figure 3:**
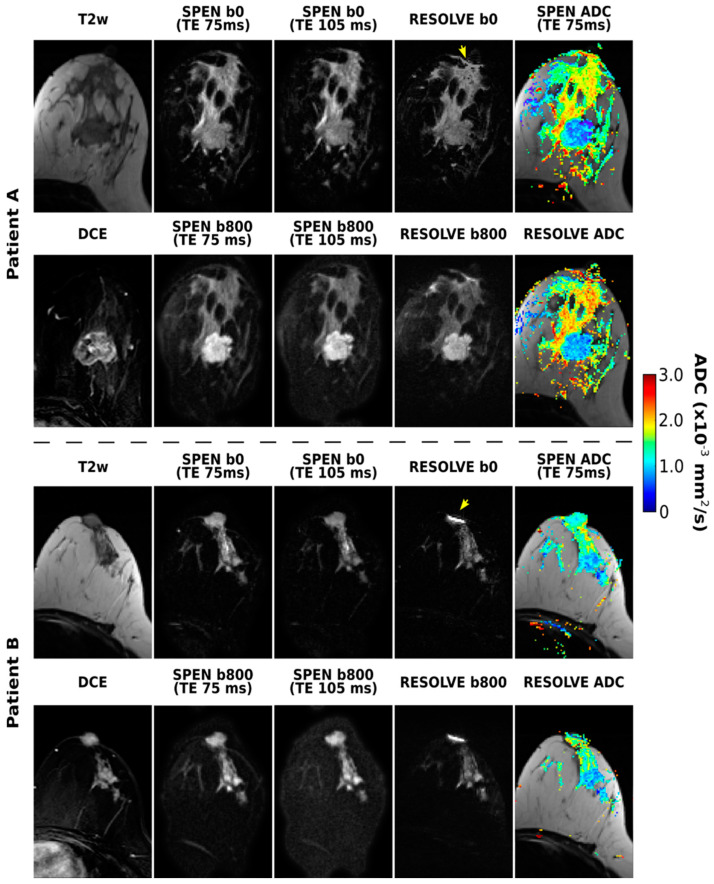
SPEN and RESOLVE acquisitions arising from b = 0 and 800 s/mm^2^ diffusion weightings, along with the resulting ADC maps, for two of the patients enrolled in the study. Also shown for completion are two DW SPEN image sets acquired with TEs of 75 and 105 ms, as well the T2w and subtracted DCE images. The TE for the RESOLVE experiments was 67 ms. Some of the distortions observed in the RESOLVE images are highlighted with yellow arrows. For Patient A (corresponding to patient 1 in Table 1), lesion borders coincide with the mass enhancement in DCE image, while for Patient B (corresponding to patient 5 in Table 1), essentially all the non-fatty tissue present in T2 image was judged to be cancerous.

The RESOLVE-based acquisitions were also considered for the purpose of this joint T2–ADC breast mapping. Figure 3 shows these results, together with equivalent SPEN data, for two of the patients enrolled in this study. RESOLVE generally shows lower immunity to susceptibility-induced artifacts, as evidenced, for instance, by pile-ups in the vicinity of the nipples. This is in contrast to the SPEN images, which deliver satisfactory SNR and good highlighting of the tumors upon DWI, without these distortions. Also worth remarking on are the somewhat lower ADC values arising from the SPEN images, which we ascribe to a slightly better robustness against motions. Notice, as well, that while noise levels increase at longer TEs, the overall image quality acquired at TE 105 ms is satisfactory even in the presence of the diffusion weighting. No differences were noticed if either b- or TE-values were sampled as the outer loop.

**Figure 4:**
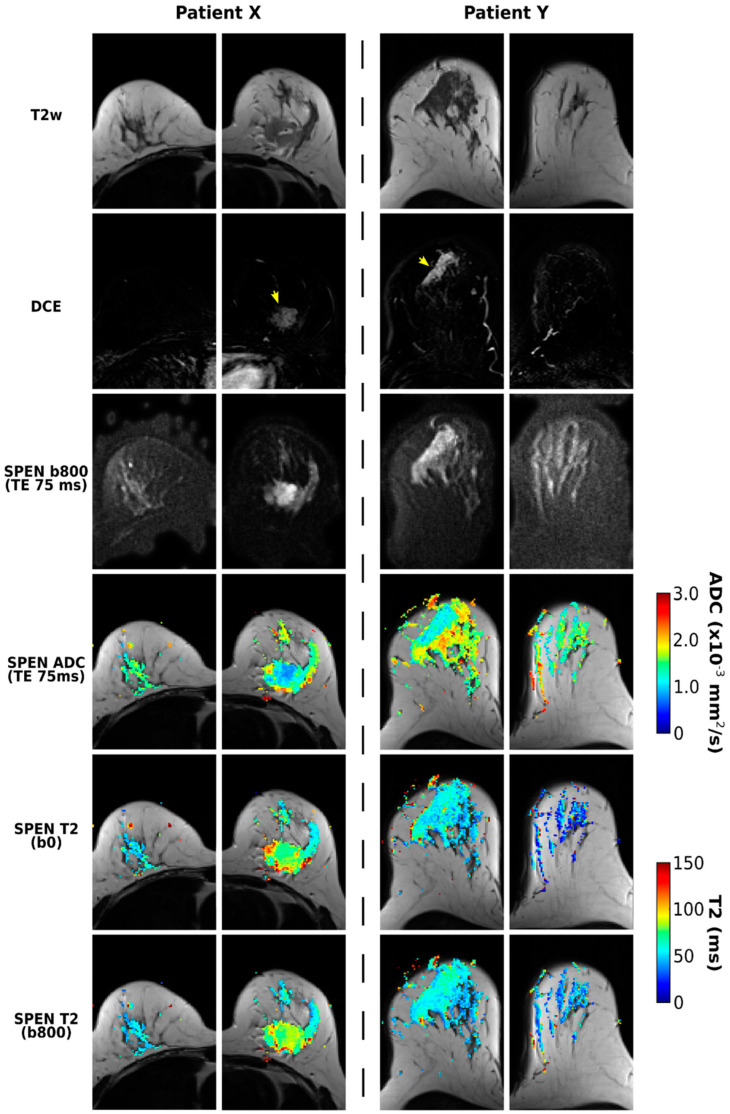
SPEN acquisitions for two patients (X and Y, corresponding to patients 12 and 7 in Table 1, respectively) diagnosed with breast carcinomas. Presented on the top rows are T2w, DCE, and DW images collected for TE = 75 ms, with the lesions labeled with yellow arrows. Shown on the lower rows are ADC maps derived from images acquired using TE = 75 ms, as well as T2 maps derived from b0 and b800 DWI data.

Figure 4 illustrates further the methodology proposed for separating and correlating ADC and T2 maps for breast cancer patients. Highlighted in these data are the quality T2 maps that can be retrieved even after the application of a relatively strong diffusion encoding. Notice that some contrast appears to arise in the T2 mapping for distinguishing malignant lesions from healthy tissue—particularly when comparing to the T2 values in the contralateral breast. Another feature arising when comparing the T2 maps calculated from b = 0 and 800 s/mm^2^ images is a certain attenuation of the longer T2 component, a feature that is particularly noticeable in the area of the lesion for Patient X.

**Figure 5:**
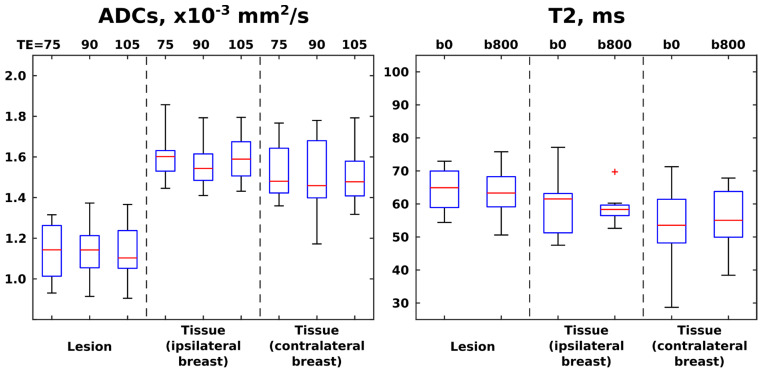
Box plots (median ± interquartile range) and whiskers (from minimum to maximum values) calculated from the ADC and T2 values derived from SPEN images acquired at different TEs (**left panel**) and b-weightings (**right panel**), respectively. The red cross denotes an outlier, here defined as a value that is more than 3 times the interquartile range away from the bottom or top of the boxes (denoting, respectively, 25th and 75th percentiles of the sample data). ADC values summarize results over 12 patients, while T2 summarize values over 11 patients. ROIs were drawn for cancerous lesions and healthy tissue, separately analyzing healthy tissue from tissue in breast ipsilateral and contralateral to the breast containing the lesion.

With these tools at hand, Figure 5 presents boxplots of the ADC and T2 values resulting from patient-oriented measurements at various TE and b-weightings, respectively. Here, each of the patients contributes a single data point per boxplot as an averaged value over the whole relevant region-of-interest (ROI); i.e., after delineating ROIs corresponding to cancerous and healthy tissues for each of the patients, mean T2 and ADC values over all of the voxels corresponding to these ROIs were calculated. Averaged ADC and T2 values over all voxels arising from slices located in the same axial plane, but in the contralateral (non-diseased) breast, are presented in these box plots as well. Contralateral breasts did not contain cancerous tissue in any of the patients, as was confirmed by examination of the DCE images. For one of the subjects, it was not possible to perform T2 mapping due to motion between the DW acquisitions using different TEs; hence the respective boxplots summarize ADC values for 12 patients and T2 values for 11 patients. It follows from these results that ADC values provide a clear diagnostic separation between healthy and cancerous tissue for every TE value. Average T2s are clearly inferior to ADCs as indicators of cancer regardless of b-weightings—even if there seems to be, on average, slightly longer T2s for the ROIs with lesions. The ADC values derived at longer TEs, as well as T2 values derived using b-weighting, provide larger scatterings vis-à-vis values derived at the shortest TE and no b-weighting, respectively. This is arguably a consequence of sensitivity penalties arising at these larger b- and TE values.

**Figure 6:**
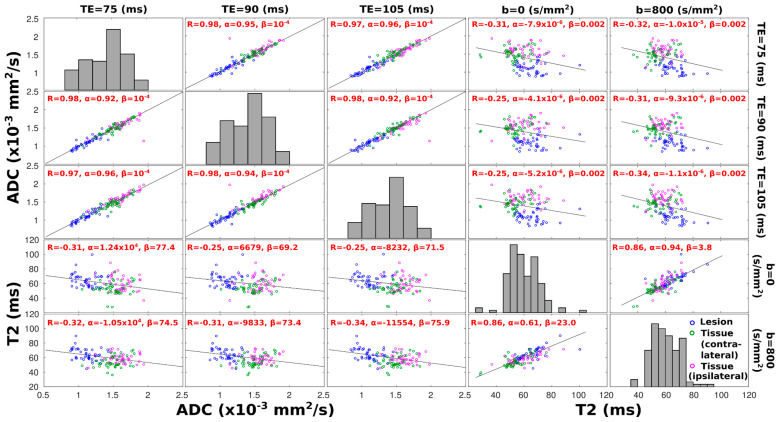
Scatter plots of mean ADC and T2 values calculated over regions corresponding to cancerous (blue dots) tissue, to healthy tissue arising from the contralateral breast (green dots), and to the ipsilateral, lesion-containing breast (pink dots). Each data point corresponds to the average value over the respective area from one slice; only data from slices containing lesions and corresponding contralateral slices are included in this analysis. For the first three columns (rows), *x*-axes (*y*-axes) describe ADC values, while for the fourth and fifth columns (rows), *x*-axes (*y*-axes) describe T2 values. Respective TE/b-values at which ADC–T2 values were derived are given on the top and right side of the plots. Diagonal subplots display a histogram of distribution of the ADC or T2 values derived at a given TE or b-value, respectively. For each off-diagonal plot at the top left corner, a correlation coefficient R is provided, along with linear regression coefficients α and β, corresponding to slope and intercept, respectively; a least-squares reference line with slope equal to the Pearson’s correlation coefficient is also drawn. All correlation coefficients display correlations that are significantly different from zero (*p*-values < 0.05).

Figure 5 presents global T2 and ADC trends with b-weighting and echo time. A clearer indicator of whether ADC and T2 values are correlated is assessed in Figure 6, which presents similar correlations but on a region-by-region analysis for the various patients, with lesion, ipsi-, and contralateral ROIs represented by different colors. The most evident feature of this analysis is the almost perfect correlation that exists between the ADC values derived at different TEs; this highlights the small influence that T2 weightings have on DWI-derived ADC values. A somewhat similar—though weaker and more scattered—correlation is observed between T2 values derived at different b-weightings; this suggests that a stronger or weaker weighting, based on water’s diffusivity, will not filter out subcomponents possessing shorter or longer T2 relaxations. Intriguingly, however, a mutually consistent anticorrelation arises between the T2 and ADC values when evaluated for all TE and b-value pairs, as evidenced by the off-diagonal top-right and bottom-left panels in Figure 6. This anticorrelation becomes stronger with an increase in diffusion weighting; a similar anticorrelation between the T2 and ADC values is observed at b = 800 s/mm^2^, if analyzing solely data points corresponding to lesions (Supplementary Figure S1). Another noticeable feature of these T2–ADC correlations is a relative clustering of data points arising from the lesions and from healthy collateral and contralateral breast tissues. For the lesion ROIs, this is expected, as their ADC values are markedly lower (Figure 5), and that will separate their data from the rest; notice, however, that data points corresponding to the healthy contralateral breasts are also clustered in both the T2–ADC and ADC–T2 correlations, falling in between data points corresponding to the lesion and data points stemming from the healthy tissue in the diseased breast.

## 4. Discussion

This study was motivated by a search for correlations between ADC and T2 values in breast cancer patients. While it is well known that ADC-based measurements can distinguish these cancerous lesions while T2 contrast is insufficient for diagnosing breast cancer [16,49,50,51], we hypothesized that the morphological tissue changes known to affect the former will also have influence on the latter. To explore this, SPEN-based protocols were developed, incorporating the option to impart correlated diffusion- and T2-weightings into the sets. No new insight was found when evaluating the ADC- or T2-based images on their own: diffusivity showed clear distinctions between cancerous and normal tissues, while relaxation failed to do so. However, small but meaningful correlations arose when considering these parameters for the different tissue types that were analyzed. These, however, were not as anticipated: conventional NMR considerations, based on decreased mobilities leading to stronger zero-frequency spectral densities, suggest that the cellular restrictions associated to lower ADC values will also shorten the water’s T2 times. This behavior has been observed in a wide variety of systems, ranging from foods and lunar samples [17,18] to brain [19] and prostate cancers [52]. This is contrary to the trends revealed by Figure 6, where sectioned tissues show an anticorrelation whereby restricted water diffusivity is accompanied by longer relaxation times. The longer T2s arising from our study for the malignant breast lesions over normal fibroglandular tissues are actually in accordance with previous literature reports. For instance, cohorts of exams on healthy volunteers reported T2 medians of 55 ± 10 and 71 ± 6 ms for fibroglandular tissues [48,53], while a recent, larger study reports 90 ± 20 ms for cancerous regions; both of these were measured at 3 T [54]. We conjecture that this T2 lengthening is caused by changes in tissue properties, which bring out factors that go beyond simple cellular restriction arguments in the definition of the actual T2s. This could include differences in intra- vs. extracellular water volumes and/or differences in the actual properties of the cells—including, for instance, a decreased viscosity, mosaicity, composition, or porosity of the membranes involved in the different tissues. Particularly interesting is the contrast revealed by the T2–ADC correlations when comparing healthy breast tissues that are ipsilateral but separate from the lesions and healthy contralateral tissues (Figure 6). Should there have been an ambiguous delineation of the lesion borders, healthy ipsilateral T2–ADC tissue correlations would have been expected to cluster in between points arising from the contralateral breast and from the lesions. By contrast, the T2–ADC correlations evidenced a clear clustering of the ipsilateral points furthest away from the lesions’ values (e.g., lower-left panels in Figure 6). This can, to some extent, also be perceived in Figure 4, where ADC and T2 maps from both breasts are presented: ADC values corresponding to healthy and cancerous tissue appear more contrasted when considered in the same breast than when considering the lesion and the contralateral breast. Likewise, the contrast in T2 values is more pronounced between cancerous and healthy tissues belonging to the same breast. All this suggests that there is an actual change in the tissue properties arising from the presence of the lesion—even in regions of the affected breast that have not yet been infiltrated. This factor may have also been an unrecognized aid when using ADC values and DWI studies to characterize these lesions.

The analysis presented herein also offers insight into the choice of experimental parameters that can maximize contrast between cancerous and healthy tissue in DWI-based analysis. We and others [35,55] have shown that high b-weighting maximizes the lesion contrast, even if, eventually, sensitivity gets too penalized. The raises the question of whether, for a given b-weighting, there is an appropriate choice of TE—i.e., of T2 weighting—that, based on the results above, can maximize the contrast between healthy and diseased tissue. Supplementary Figure S2 analyzes how the contrast-to-noise ratio (CNR) of these two tissues changes as a function TE, assuming the average T2 values deriving from Figure 6. Disregarding the sensitivity’s dependence on TE—admittedly, a fairly strong assumption—it follows that an optimum TE for adding T2 effects to the differentiation between cancerous and healthy tissues is obtained at TE ≈ 59 ms (Figure S2). This CNR curve has a relatively broad plateau, meaning that deviations of ±20 ms will affect this T2-derived contrast-to-noise ratio by ≤5%.

## 5. Conclusions

This study employed accelerated SPEN-based image acquisition protocols for encoding T2 and ADC values on breast cancer patients at 1.2 × 1.2 mm^2^ in-plane resolutions. The acquisition and processing strategies provided sufficiently high immunity to motion and inhomogeneity artifacts to enable the acquisition of reliable T2 and ADC maps, as well as to establish T2–ADC correlations. T2 and ADC behaviors of healthy and diseased tissues were as expected, but the correlation observed for these two parameters was consistently opposed to what naïve considerations based on water restrictions would have suggested. We hypothesize that these reflect actual changes in either intracellular/extracellular ratios and/or on actual cellular morphologies associated to changes in the membrane or other macromolecular components of the tissues. Extension of this study to incorporate minimally invasive ways of assessing these properties by non-MRI methods are being considered. The weak but systematic correlations of the derived T2–ADC values also suggests that these parameters contain some complementary information, and that their combined use could assist in improved breast lesion characterization.

Despite the above-mentioned conclusions, this study also has a number of limitations. The relatively small number of patients that was scanned may have limited the statistical relevance of the results. In addition, the studied cohort included a range of breast cancer pathologies with several molecular subtypes; hormonal status within the cohort—which included both young volunteers during the menstrual cycle and post-menopausal volunteers—was also left unchecked. Future studies involving a larger cohort of patients, including those with benign tumors, and relying on a more controlled set of tumor and hormonal characteristics, will allow us to broaden the validity of our results and further investigate the quantitative ADC–T2 correlation in the various subtypes of breast cancer.

## Figures and Tables

**Table 1:** Characteristics of the consenting patients examined in this study. Abbreviations: DCIS, Ductal carcinoma in situ; ER, estrogen receptors; HER2, human epidermal growth factor receptor 2; IDC, invasive ductal carcinoma; ILC, invasive lobular carcinoma; PR, progesterone receptors.

Patient	Age	Size on DCE Images (Largest Diameter), cm	Pathology
1	33	3.5	IDC triple negative
2	45	2.5	IDC triple negative
3	46	1	IDC ER+ PR+ HER2−
4	48	1	ILC ER+ PR+ HER2−
5	27	1.7	IDC+ DCIS ER+ PR+ HER2−
6	66	1.4	IDC ER+ PR+ HER+
7	34	4.6	IDC ER+ PR+ HER2−
8	39	0.9	DCIS ER+ PR+ HER2−
9	52	3.1	IDC triple negative
10	41	1.5	DCIS ER− PR− HER2+
11	46	1.3	IDC ER+ PR+ HER2−
12	46	3.2	IDC triple negative

## Data Availability

Data published in this study is available from the authors upon reasonable request.

## Supplementary Materials

The following supporting information can be downloaded at: https://www.mdpi.com/article/10.3390/diagnostics13233516/s1, Figure S1: Scatter plots of mean ADC and T2 values calculated over regions corresponding to cancerous lesions; Figure S2: Signal intensity dependence as a function of TE for typical T2 values corresponding to cancerous lesion and to healthy tissue.

## Abbreviations

ADC, apparent diffusion coefficient; DCE, dynamic contrast enhanced; DCIS, ductal carcinoma in situ; DWI, diffusion-weighted imaging; ER, estrogen receptors; EPI, echo-planar imaging; HER2, human epidermal growth factor receptor 2; IDC, invasive ductal carcinoma; ILC, invasive lobular carcinoma; PE, phase encoding; PR, progesterone receptors; RESOLVE, readout segmentation of long variable echo-trains; SPEN, spatiotemporal encoding; SNR, signal to noise ratio.
