# Supplementary material for: Relaxation–Diffusion T2–ADC Correlations in Breast Cancer Patients: A Spatiotemporally Encoded 3T MRI Assessment
Source: Diagnostics (Basel). 2023 Nov 23;13(23):3516. doi: 10.3390/diagnostics13233516 (PMC10705897; doi:10.3390/diagnostics13233516)
Supplement: Supplementary file 1 [file diagnostics-13-03516-s001.zip › diagnostics-2688335-supplementary.pdf]

Supporting Information

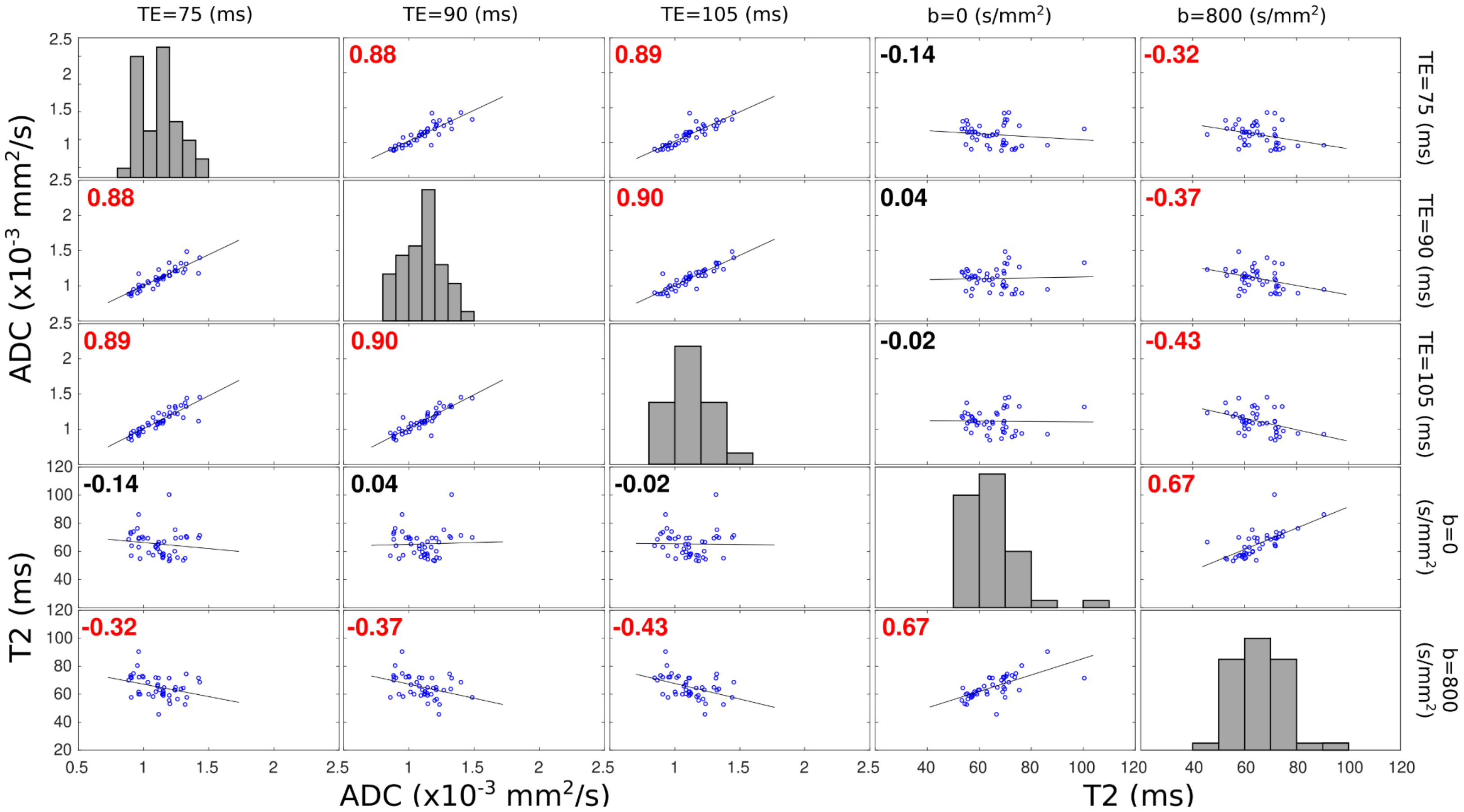

Fig. S1. Scatter plots of mean ADC and T2 values calculated over regions corresponding to cancerous lesions. Each data point corresponds to the average value over the respective area from one slice. For the first three columns (rows), x-axes (y-axes) describe ADC values, while for the fourth and fifth columns (rows), x-axes (y-axes) describe T2 values. Respective TE / b-values at which ADCs / T2s were derived, are given on the top and the right side of the plots. Diagonal subplots display a histogram of distribution of the ADC or T2 values derived at a given TE or b-value, respectively. For each off-diagonal plot, Pearson correlation coefficients are provided and a least-squares reference line with slope equal to Pearson's correlation coefficient is also drawn. Pearson coefficients displayed in red denote significant correlations with *p*-values < 0.05; coefficients in black denote no significant correlation.

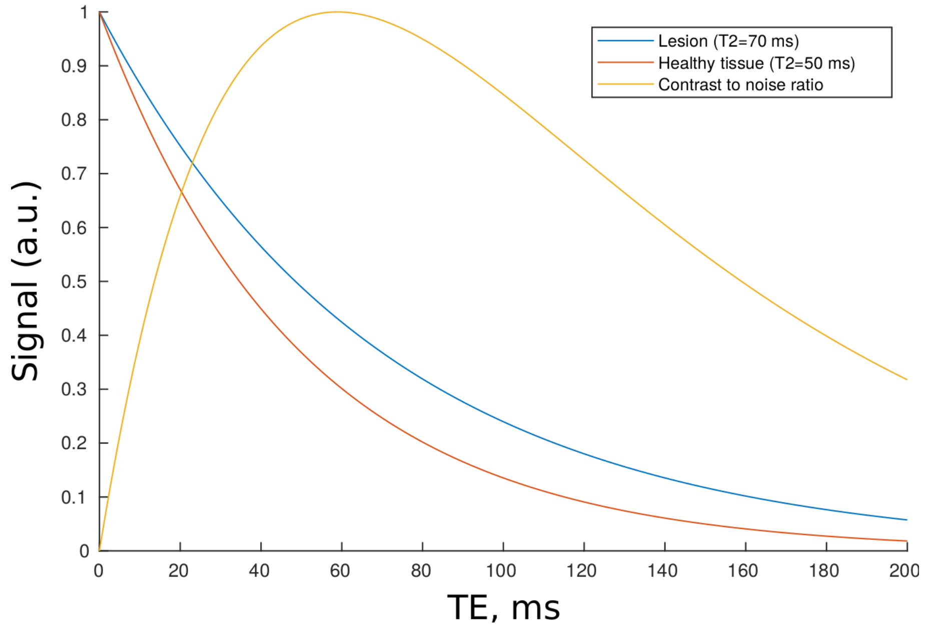

Fig. S2. Signal intensity dependence as a function of TE for typical T2 values corresponding to a cancerous lesion (70 ms, blue curve) and to a healthy tissue (50 ms, red curve), as described by equation 4 in the main text along with respective contrast-to-noise ratio's dependence on TE (yellow curve), corresponding to normalized difference of the two signal intensity curves. Constant noise level was assumed for all the TEs.
